# Diagnostic Value of Combined Biomarkers and the Shock Index for Predicting Febrile Neutropenia and Clinical Outcomes in the Emergency Department: A Multivariate Regression Analysis

**DOI:** 10.3390/jcm15218234

**Published:** 2026-10-22

**Authors:** Murat Muratoglu, Busra Bulut, Saide Bakal Muratoglu, Mohammadsajad Cheraqi, Fatih Yıldırım

**Affiliations:** 1 Department of Emergency Medicine, Faculty of Medicine, Başkent University, Ankara 06490, Türkiye; mdcldctr@gmail.com (B.B.); mcheraqi@baskent.edu.tr (M.C.); fatihyildirim2053@gmail.com (F.Y.); 2 Department of Physiology, Faculty of Medicine, Yüksek İhtisas University, Ankara 06530, Türkiye; saide.bakal@hotmail.com

**Keywords:** febrile neutropenia, neutrophil-to-lymphocyte ratio, shock index, oncologic emergencies, risk stratification, multivariable regression analysis

## Abstract

**Background**: Early risk stratification of febrile neutropenia (FN) in the emergency department (ED) is vital to accelerate empirical antibiotic management. This study evaluates the independent diagnostic performance of combined blood-borne inflammatory indices and the Shock Index (SI) in predicting FN status and severe clinical outcomes. **Methods**: Medical records of 338 febrile oncology patients presenting to the ED between 2020 and 2026 were retrospectively evaluated. Patients were divided into the FN group (fever + ANC < 1000 cells/μL; *n* = 213) and the control group (*n* = 125). Advanced cell-line metrics and biochemical ratios were calculated at admission. Independent predictors were isolated using multivariable logistic regression analysis. **Results**: Multivariable regression analysis revealed that elevated core body temperature (aOR: 3.229, 95% CI: 1.845–5.652, *p* < 0.001), lower serum sodium levels (aOR: 0.843, 95% CI: 0.767–0.926, *p* < 0.001), a low Neutrophil-to-Lymphocyte Ratio (NLR) (aOR: 0.636, 95% CI: 0.549–0.736, *p* < 0.001), and an elevated Shock Index (aOR: 24.010, 95% CI: 2.258–255.360, *p* = 0.008) were stable independent predictors of FN. The combined four-variable integrated model achieved an outstanding Area Under the Curve (AUC) of 0.906 (95% CI: 0.874–0.937) with a diagnostic sensitivity of 77.4% and a specificity of 87.0%. **Conclusions**: Integrating baseline core temperature, hyponatremia, low cellular NLR, and physiologic SI values creates a rapid, cost-effective, and highly accurate screening platform to optimize emergency triage workflows and prevent computational delivery latency in critical oncologic emergencies.

## 1. Introduction

Neutropenic fever and neutropenic sepsis are critical, life-threatening oncological emergencies in oncology patients receiving cytotoxic chemotherapy, with high morbidity and mortality rates ranging from 2% to 21% [1,2]. Due to severe immunosuppression in these patients, bacterial infections can rapidly progress to systemic collapse without demonstrating classic localized inflammatory findings [1]. Therefore, initiation of empirical broad-spectrum antibiotic treatment within the first hour of admission to the emergency department (ED) (door-to-needle time < 60 min) is the most fundamental clinical parameter determining patient survival [3].

In clinical practice, the *Multinational Association for Supportive Care in Cancer* (MASCC) and the *Clinical Index of Stable Febrile Neutropenia* (CISNE) scoring systems are widely used to provide risk stratification in ED triage [4,5]. However, since these traditional systems are largely based on the patient’s existing comorbidities, subjective symptoms, and static hemodynamic thresholds, they may be insufficient to capture the magnitude of the acute systemic inflammatory response in real time [5]. Additionally, the deficiency of functional cellular immune components in the neutropenic population leads to delays in the early clinical detection of sepsis in ED settings, masking classic signs of infection [2,5].

To overcome these diagnostic limitations, objective and low-cost combined inflammatory indices derived from routine complete blood count (CBC) parameters and basic biochemical variables have come to the fore in recent years. In this context, cellular and metabolic markers, such as the systemic immune–inflammation index (SII), C-reactive protein/albumin ratio (CAR) and lactate/albumin ratio (LAR), have been shown to exhibit strong prognostic performance in predicting general sepsis conditions, tissue hypoperfusion, and the need for intensive care unit (ICU) admission [6,7]. Therefore, this study aims to investigate the independent diagnostic accuracy of these novel laboratory-based inflammatory indices and the physiological shock index (SI) in predicting neutropenic sepsis and adverse clinical outcomes, as well as their potential to guide clinical decisions, in oncology patients presenting to the ED with neutropenic fever.

## 2. Materials and Methods

### 2.1. Design and Setting of the Study

Using an observational cohort design, the present study retrospectively examined the medical records of oncology patients presenting to the Adult ED of the Ankara Hospital at Baskent University, Türkiye between 1 March 2020 and 1 March 2026. The study protocol was approved by the Institutional Review Board and Ethics Committee of Baskent University in accordance with the principles of the Declaration of Helsinki and its 2013 amendments (Project No: KA26/135). Patient consent was waived by the ethics committee due to the retrospective nature of the study.

### 2.2. Selection of Participants and Criteria for Classification

Inclusion criteria for the study were as follows: ≥18 years of age; an active diagnosis of malignancy and receiving or having previously received cytotoxic chemotherapy; and documented objective high fever (axillary or tympanic body temperature ≥ 38.0 °C in a single measurement) at the time of ED admission or within the first 24 h of ED follow-up.

Exclusion criteria for the study were as follows: <18 years of age and the development of neutropenia being clearly indicated in the Hospital Information Management System (HIMS) records as secondary to acute SARS-CoV-2 (COVID-19) or other documented primary viral infections.

Consequently, a total of 338 patients meeting the inclusion criteria were assigned to two operational groups:

**1. The Febrile Neutropenia (Study) Group (*n* = 213):** Oncology patients previously receiving chemotherapy, presenting to the ED with high fever, and having an Absolute Neutrophil Count (ANC) < 1000 cells/µL (<1.0 × 10^3^/µL) in laboratory analysis.

**2. The Control Group (*n* = 125):** Oncology patients who had previously received chemotherapy, presenting to the ED with a high fever, but having an ANC of ≥1000 cells/µL (≥1.0 × 10^3^/µL).

We operationally defined febrile neutropenia as a single oral or axillary temperature of 38.0 C with an absolute neutrophil count (ANC) < 1000 cells/µL. Although formal ASCO/IDSA guidelines strictly emphasize an ANC < 500 cells/µL (or a predicted drop below 500 cells/µL), we selected the ANC < 1000 cells/µL threshold (corresponding to Grade 3 or higher neutropenia according to the Common Terminology Criteria for Adverse Events [NCI-CTCAE v5.0]) to capture both established severe neutropenia and evolving clinical deterioration in the emergency setting, where rapid decisions about initiating empirical antibiotics are required [8,9].

### 2.3. Data Accumulation Period and Descriptions of Variables

Demographic, clinical, and laboratory parameters of patients were retrospectively recorded via HIMS at the time of initial admission to the ED, immediately following triage assessment and admission to the treatment cabinet, and in the initial phase before any empirical *IV* antibiotic treatment or aggressive *IV* fluid resuscitation was initiated in the ED. All laboratory data used in the analyses were obtained from the initial venous and arterial blood samples drawn before treatment to clearly reflect the initial biological status of the patient in the ED. The recorded variables consisted of age, sex, vital signs on admission [systolic blood pressure (SBP), pulse rate], laboratory parameters [leukocyte (WBC), erythrocyte (RBC), platelet (Plt), absolute neutrophil (ANC), absolute lymphocyte (ALC) and absolute monocyte (AMC) counts from CBC; serum sodium (Na), glucose, power of hydrogen (pH) and blood lactate (LAC) levels] and acute-phase reactants [C-reactive protein (CRP) and procalcitonin (PCT)]. In line with the literature, PCT values ≥ 0.25 µg/L were considered positive (indicator of high metabolic risk) [5].

### 2.4. Outcomes of the Study

**Primary Outcomes:** The independent prediction of febrile neutropenia status based on data at the time of admission in patients presenting to the ED with a febrile oncological condition.

**Secondary Outcomes:** The evaluation of the clinical relationships between the developed model and composite inflammatory indices and hospital admission requirements, ICU admission rates, development of septic shock, and gate-ion (first-hour antibiotic) administration times.

### 2.5. Calculation of Inflammatory Indices and Shock Index

Using initial laboratory and vital signs, all composite indices and hemodynamic indicators were calculated using the following formulas and standard clinical units:

**Shock index (SI):** heart rate (bpm)/SBP (mmHg);

**Neutrophil-to-lymphocyte ratio (NLR):** ANC (×10^3^/µL)/ALC (×10^3^/µL);

**Lymphocyte-to-monocyte ratio (LMR):** ALC (×10^3^/µL)/AMC (×10^3^/µL);

**Systemic immune–inflammation index (SII):** [Plt count (×10^3^/µL) × neutrophil count (×10^3^/µL)]/lymphocyte count (×10^3^/µL);

**C-reactive protein-to-albumin ratio (CAR):** serum CRP (mg/L)/serum albumin (g/dL);

**Lactate-to-albumin ratio (LAR):** LAC (mmol/L)/serum albumin (g/dL);

**Fibrinogen-to-albumin ratio (FAR):** plasma fibrinogen (mg/dL)/serum albumin (g/dL).

### 2.6. Statistical Analysis

Statistical analyses were conducted using Python version 3.12 (Python Software Foundation, Beaverton, OR, USA). We assessed normality of continuous data using the Shapiro–Wilk test, histograms, and Q–Q plots. We present normally distributed continuous data as mean ± standard deviation (SD), and non-normally distributed data as median and interquartile range (IQR). Categorical variables are summarized with frequencies and percentages (*n*, %). For group comparisons, we used the independent-samples t-test for normally distributed continuous variables, the Mann–Whitney U test for non-normally distributed continuous variables, and the Pearson chi-square or Fisher’s exact test for categorical variables.

We conducted multivariable logistic regression analyses to identify variables independently associated with febrile neutropenia. To enhance analytical power and prevent information loss during model specification, age, body temperature, systolic blood pressure (SBP), serum sodium (Na) levels, blood lactate (LAC) concentration, and all calculated composite inflammatory indices (NLR, SII, CAR, LAR, FAR, and LMR) were included as continuous variables. Sex, the need for hospitalization, admission to the intensive care unit (ICU), and the development of septic shock were coded as categorical variables. Before analysis, we examined the distribution of missing data; the proportion was less than 2.0% of the total dataset, and the data were missing completely at random (MCAR). To preserve data integrity and avoid introducing artificial bias, we did not use imputation methods for missing data; instead, we adopted a “complete case analysis” approach, excluding the limited number of patients with missing data from the analysis.

Before constructing the multivariable model, we rigorously assessed multicollinearity among continuous variable candidates—identified as having *p* < 0.10 in univariable analyses—using Spearman correlation coefficients and the variance inflation factor (VIF). In cases where pairs of variables exhibited strong multicollinearity (VIF > 5.0 or r > 0.70)—such as between SII and NLR—the variable deemed more clinically stable and with a lower VIF (in this instance, NLR) was selected for inclusion in the model.

To identify the final independent predictors, we used a “backward stepwise” multivariable logistic regression approach (with *p* = 0.05 for entry into the model and *p* = 0.10 for removal). The primary rationale for choosing the backward stepwise method—as opposed to forward selection—is its ability to incorporate all candidate variables from the outset, thereby fully accounting for complex, simultaneous interactions and suppressor effects among the variables, which minimizes the risk of a Type II error. Using this model, we calculated adjusted odds ratios (aORs) with 95% confidence intervals (CIs). We evaluated the discriminative diagnostic performance of the final multivariable probability model using receiver operating characteristic (ROC) curve analysis and the area under the curve (AUC). Model calibration was assessed using calibration plots and the Brier score. We performed internal validation using 350 patient-level bootstrap resamples and calculated an optimism-adjusted AUC. We determined the optimal probability threshold using the Youden Index; we then calculated the model’s diagnostic sensitivity, specificity, and overall classification accuracy based on this threshold. We examined factors associated with intensive care unit (ICU) admission, septic shock, and 30-day mortality within the febrile neutropenia (FN) group using separate multivariable logistic regression models. We evaluated the MASCC score in eligible patients with febrile neutropenia. All statistical tests were two-tailed, and we considered *p* < 0.05 statistically significant.

## 3. Results

A total of 338 participants were included in the study, comprising 213 patients with febrile neutropenia and 125 control subjects.

The demographic and clinical characteristics of the study population are presented in Table 1. The median age was comparable between the febrile neutropenia and control groups [68 (IQR: 56–74) vs. 68 (IQR: 58–74) years, *p* = 0.711].

Patients with febrile neutropenia were significantly more likely to receive antibiotic therapy than controls (57.7% vs. 20.8%, *p* < 0.001). Among patients who received antibiotics, the proportion receiving treatment within the first hour was similar between groups (33.3% vs. 34.6%, *p* = 1.000). Hospital admission (65.3% vs. 52.8%, *p* = 0.032), ICU admission (22.5% vs. 10.4%, *p* = 0.008), and septic shock (21.1% vs. 8.8%, *p* = 0.005) were significantly more frequent in the febrile neutropenia group.

Comparisons of vital signs are summarized in Table 2. Patients with febrile neutropenia had significantly higher body temperature [37.10 (36.50–38.10) °C vs. 36.70 (36.30–37.10) °C, *p* < 0.001] and LAC levels [1.70 (1.30–2.42) mmol/L vs. 1.50 (1.00–2.20) mmol/L, *p* = 0.014]. SBP was significantly lower in the febrile neutropenia group (122.59 ± 20.74 vs. 129.60 ± 20.01 mmHg, *p* = 0.002), whereas blood pH was also slightly lower [7.39 (7.35–7.45) vs. 7.41 (7.37–7.45), *p* = 0.049].

Laboratory findings are presented in Table 3. Compared with controls, patients with febrile neutropenia had significantly higher CRP levels (*p* < 0.001) and lower sodium concentrations (*p* = 0.005). Marked reductions were observed in RBC, WBC, PLT count, NEU count, and LYMPH count (all *p* < 0.001). The MONO count was also significantly lower in the febrile neutropenia group (*p* < 0.001). The PCT levels were significantly higher in patients with febrile neutropenia than in controls (*p* = 0.008).

Comparisons of inflammatory indices are shown in Table 4. Significant differences were observed for NLR, LMR, SII, CAR, LAR, and SI (all *p* < 0.05). Specifically, patients with febrile neutropenia exhibited lower NLR and SII values, whereas CAR, LAR, LMR, and SI values were significantly higher than those of the control group. In contrast, the platelet-to-lymphocyte ratio and FAR were comparable between groups (*p* = 0.220 and *p* = 0.138, respectively).

**Figure 1:**
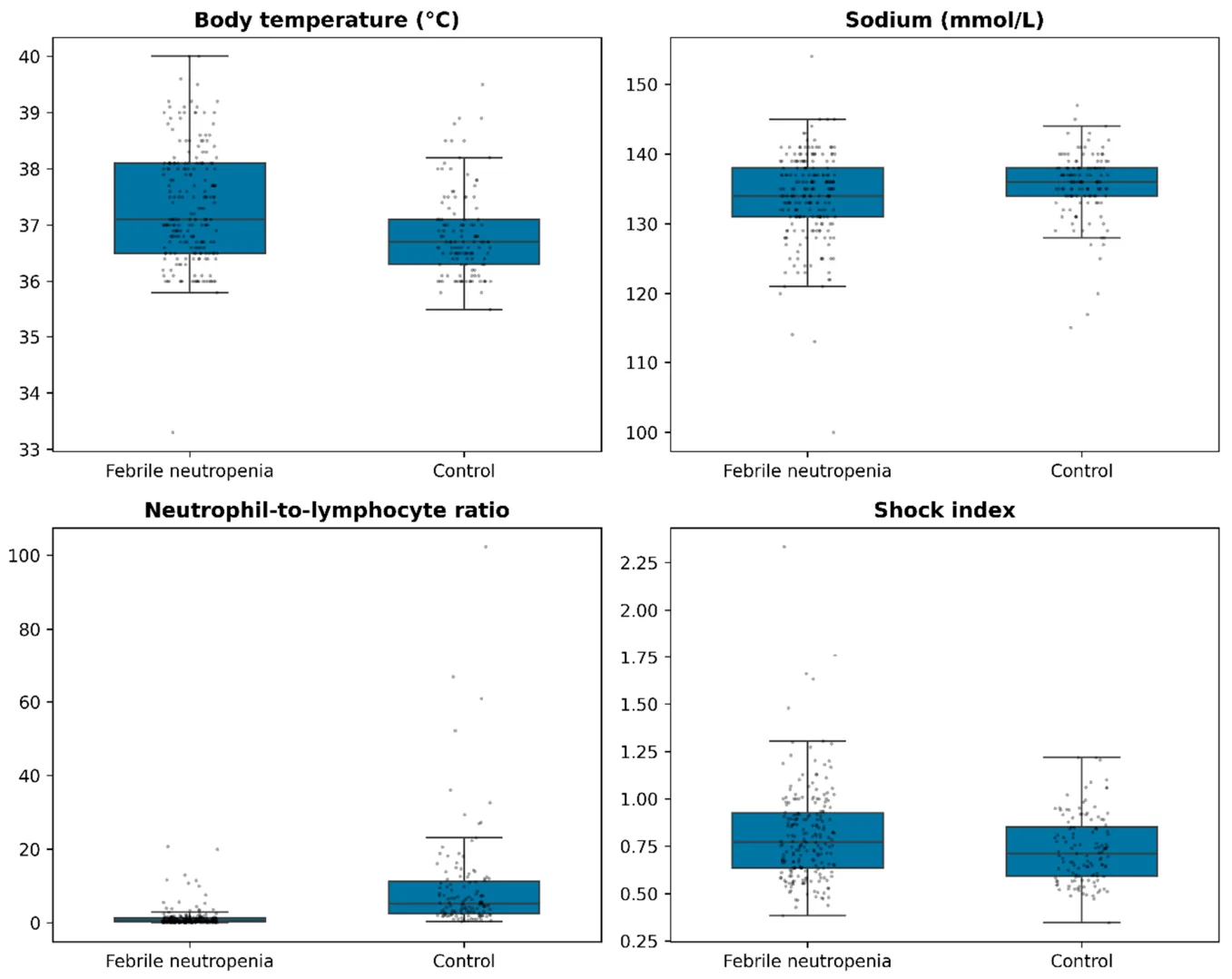
Boxplots of the final model variables in patients with febrile neutropenia and controls.

The distribution of the variables retained in the final multivariable logistic regression model is presented in Figure 1. Patients with febrile neutropenia exhibited higher body temperature and SI values, lower sodium levels, and markedly lower neutrophil-to-lymphocyte ratio (NLR) values compared with the control group. These visual findings are consistent with the results of the univariate analyses.

**Multivariable Model and ROC Analysis**

Variables with *p* < 0.10 in the univariate analysis were entered into a multivariable logistic regression model. Following backward elimination, four variables remained independently associated with febrile neutropenia (Table 5).

The four-variable model was evaluated using data from 338 presentations of 192 patients (213 FN cases, 125 controls). In multivariable analysis, body temperature (aOR = 2.25 per 1 °C increase; 95% CI: 1.50–3.37; *p* < 0.001), serum sodium (aOR = 0.87 per 1 mmol/L increase; 95% CI: 0.82–0.92; *p* < 0.001), NLR (aOR = 0.63 per 1-unit increase; 95% CI: 0.52–0.76; *p* < 0.001), and shock index (aOR = 1.25 per 0.10-unit increase; 95% CI: 1.09–1.43; *p* = 0.001) were independently associated with FN classification (Table 5).

**Figure 2:**
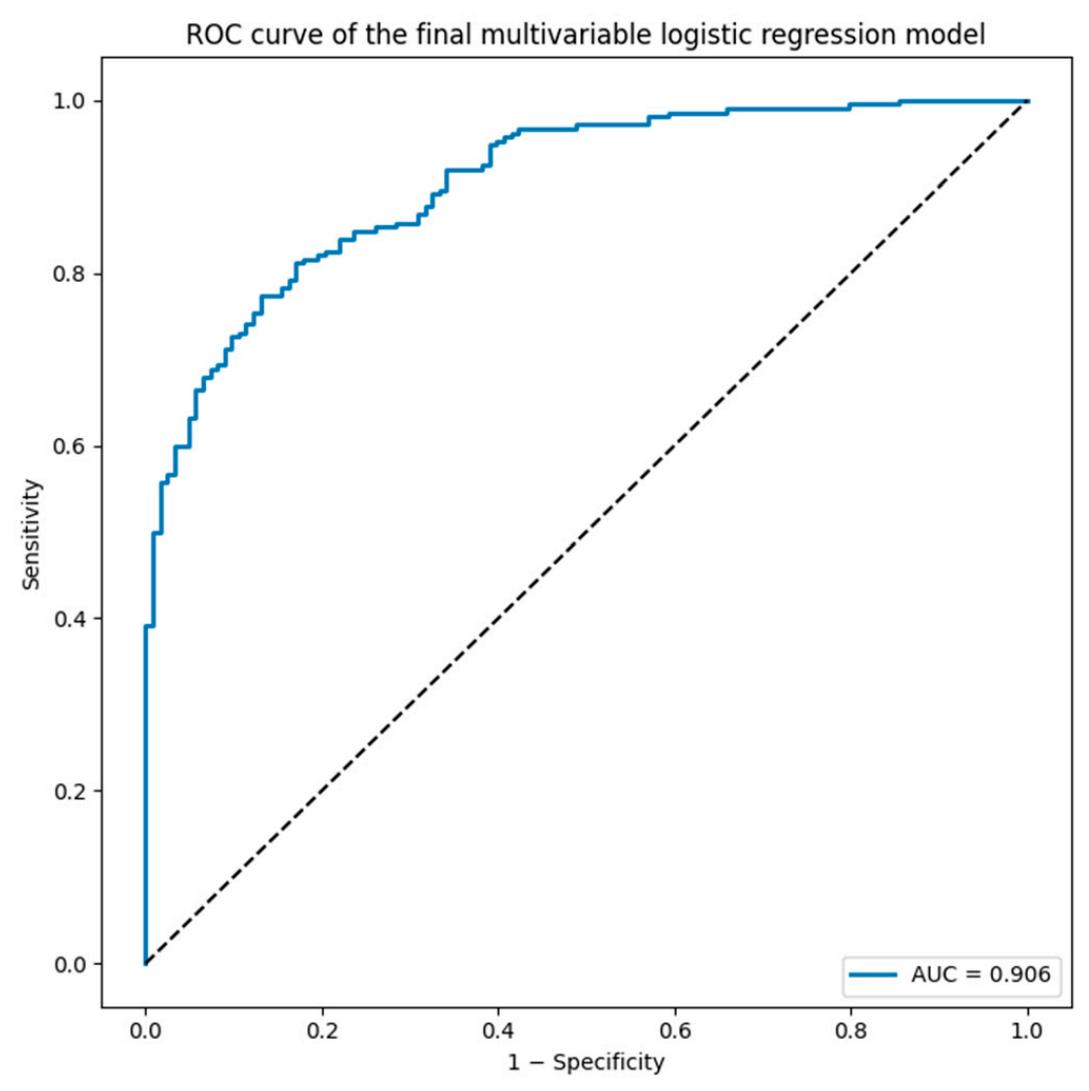
Receiver operating characteristic (ROC) curve of the final multivariable logistic regression model for identifying febrile neutropenia.

We evaluated the diagnostic performance of the final multivariable logistic regression model using receiver operating characteristic (ROC) curve analysis (Figure 2, Table 5 and Table 6).

**Figure 3:**
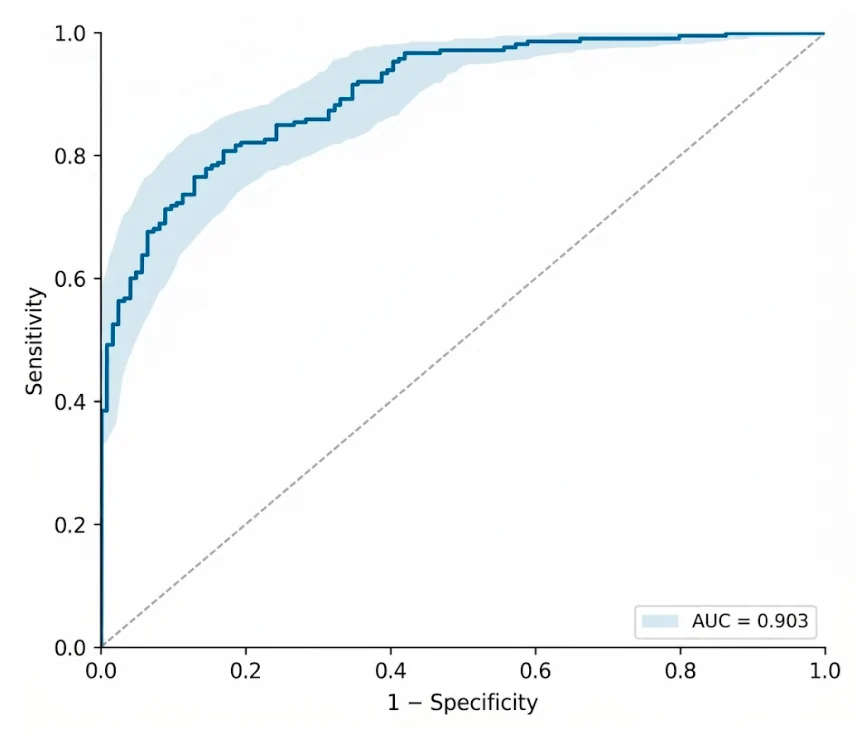
ROC curve of the four-variable model. The shaded area represents the 95% uncertainty interval obtained from patient-level clustered resampling.

**Figure 4:**
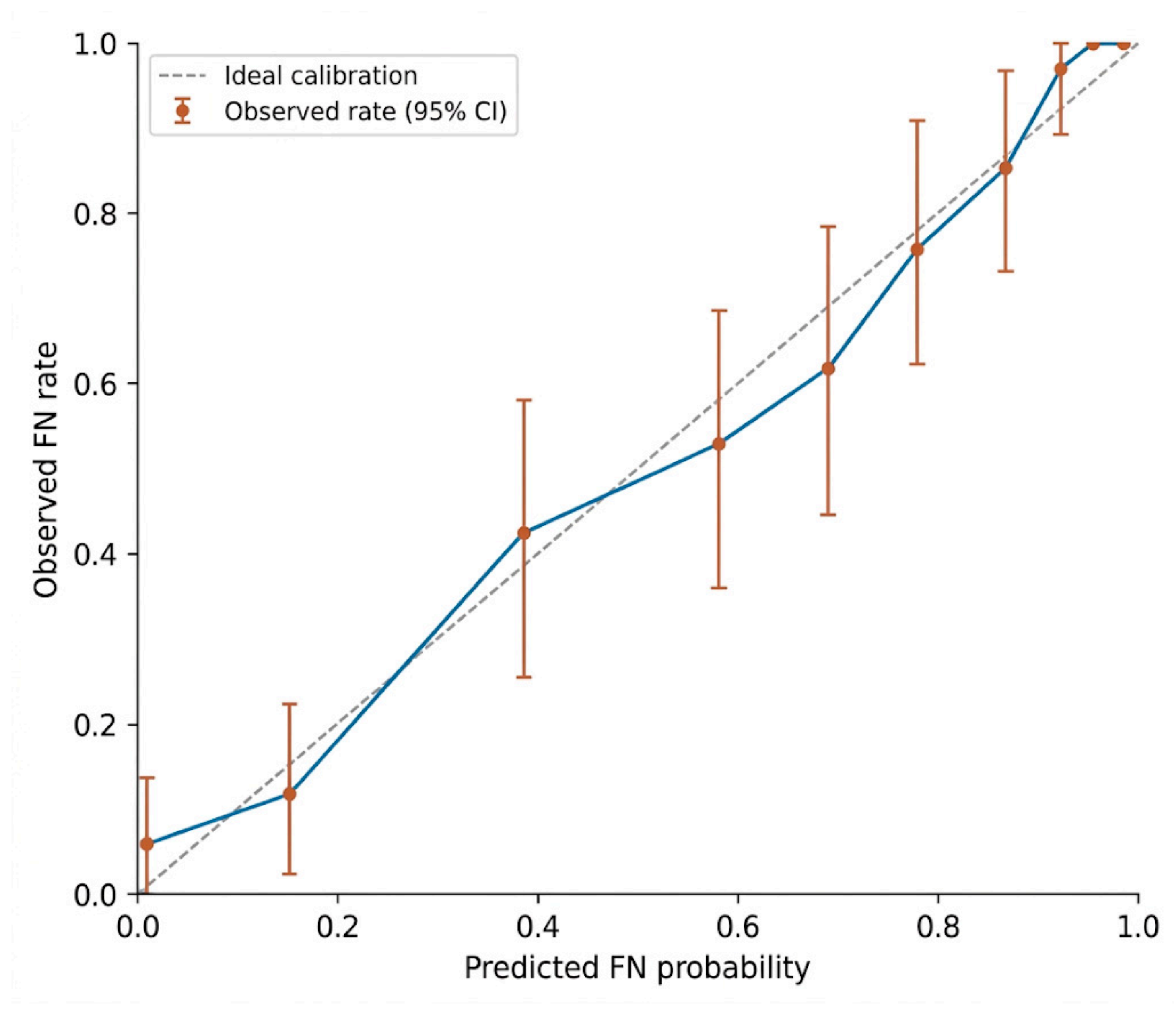
Calibration plot for FN classification. Grouped predictions and observed FN rates; vertical lines show 95% CI obtained by patient-level bootstrapping.

The model’s apparent AUC was 0.903 (95% CI: 0.871–0.932), and the optimism-corrected AUC was 0.898. The apparent and optimism-corrected Brier scores were 0.122 and 0.127, respectively. At a probability threshold of 0.50, the model achieved a sensitivity of 91.55%, a specificity of 65.32%, a positive predictive value of 81.93%, and a negative predictive value of 81.82% (Table 6; Figure 3 and Figure 4).

**Clinical Outcomes and MASCC**

**Figure 5:**
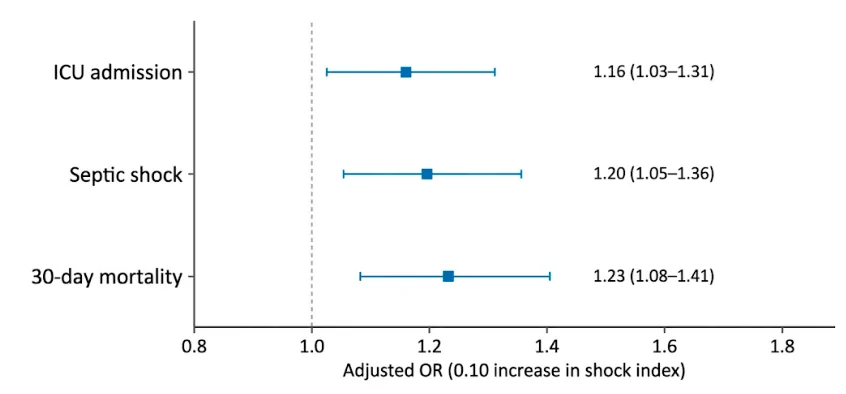
Association of shock index with clinical outcomes. aOR and 95% CI are shown per 0.10–unit increase in shock index.

Among FN presentations, the intensive care unit admission rate was 48/213 (22.54%), the septic shock rate was 45/213 (21.13%), and the 30-day mortality rate was 42/213 (19.72%). After adjusting for body temperature, serum sodium, and NLR, each 0.10-unit increase in shock index was independently associated with intensive care unit admission (aOR = 1.16; 95% CI: 1.03–1.31; *p* = 0.017), septic shock (aOR = 1.20; 95% CI: 1.05–1.36; *p* = 0.005), and 30-day mortality (aOR = 1.23; 95% CI: 1.08–1.41; *p* = 0.002) (Table 7; Figure 5). Of the 157 FN presentations with underlying malignancy, 101 had a MASCC score < 21 and 56 had a MASCC score ≥ 21. The apparent AUC values of the MASCC score for intensive care unit admission, septic shock, and 30-day mortality were 0.883, 0.850, and 0.805, respectively (Table 8 and Table 9).

MASCC: B + 5·I(systolic blood pressure > 90 mmHg) + 4·I(no COPD) + 4·I(solid tumor or hematologic malignancy with no prior invasive fungal infection) + 3·I(no dehydration requiring IV fluids) + 3·I(fever onset outside the hospital) + 2·I(age < 60 years).

CISNE: 2·I(ECOG ≥ 2) + 2·I(hyperglycemia) + I(presence of COPD) + I(presence of chronic cardiovascular disease) + I(mucositis grade ≥ 2) + I(monocytes < 0.20 × 10^3^/µL).

B = 5 (absent/mild symptoms), 3 (moderate), or 0 (severe/shock); I(condition) = 1 if the condition is met, 0 otherwise. For the CISNE score, hyperglycemia was assessed using a threshold of ≥121 mg/dL for individuals without a history of diabetes and ≥250 mg/dL for those with diabetes; the use of systemic steroids was not confirmed. The total score range is 0–26 for the MASCC score and 0–8 for the CISNE score.

## 4. Discussion

Neutropenic fever and neutropenic sepsis are oncological emergencies with high morbidity and mortality in oncology patients receiving cytotoxic chemotherapy, requiring urgent clinical intervention [2,7,10]. Rapid and accurate risk stratification of these patients upon presentation to the ED is of critical importance for the prompt initiation of empirical antibiotic treatment and early identification of those requiring admission to the ICU [2,10,11]. However, the suppression of classic signs of infection and inflammation due to insufficient cellular immune response makes clinical detection difficult [2,12]. In the present study, the prognostic values of clinical parameters, electrolyte disturbances, and inflammatory indices derived from CBC and biochemical parameters in predicting the presence and clinical severity of febrile neutropenia in oncology patients presenting to the ED were evaluated, and a multivariate model with high diagnostic accuracy was created.

Regarding clinical workflow, it is important to clarify the operational sequence of our proposed model in the emergency department. Because NLR calculation depends on automated complete blood count (CBC) reporting, this model is not designed as a pre-laboratory triage tool. Instead, its clinical utility lies in post-diagnostic risk stratification immediately after CBC results are available. Once neutropenia is laboratory-confirmed, relying solely on absolute neutrophil thresholds provides limited prognostic discrimination regarding patient trajectory. By integrating NLR alongside systemic parameters at the point of initial CBC availability, the model serves as an automated clinical decision-support trigger—aiding emergency clinicians in deciding patient disposition, early escalation of empiric therapy, or safe outpatient management.

One of the key findings of our study is that the processes of sepsis, septic shock, and organ failure progressed significantly more severely in the febrile neutropenia group. The rate of empirical antibiotic treatment was significantly higher in patients with febrile neutropenia than in the control group. Among patients receiving antibiotics, the rate of those having access to treatment within the first critical hour (door-to-needle time < 60 min) was found to be similar in both groups. In contrast to this finding, the need for general hospitalization, admission to ICU, and the development of acute septic shock were observed at a significantly higher frequency in the febrile neutropenia group. These data demonstrate how quickly the lack of baseline immune protection in neutropenic patients can deteriorate the clinical picture and strongly support similar findings in the literature [2,3,13].

Among traditional acute-phase reactants and laboratory biomarkers, CRP and PCT levels were significantly higher in the febrile neutropenia group compared with controls. PCT is a reliable marker that is not affected by neutropenia, especially in bacterial infections and in predicting sepsis [14]. In our study, however, inflammatory indices that combined cellular and biochemical parameters—namely CAR, LAR, and SI—were significantly higher in the febrile neutropenia group. In the general sepsis literature, increases in CAR and LAR levels are directly related to the inflammatory load in the body, vascular permeability, and tissue hypoxia/organ perfusion disorder, and they yield better outcomes in predicting mortality than the individual components alone [7,15].

From the perspective of cellular inflammatory indices, increased NLR and SII are associated with poor prognosis and infection in sepsis and in the general population. However, in our study, the NLR and SII values were significantly lower in the febrile neutropenia group than in the controls, which is logical given the pathophysiology of neutropenic patients, even if it seems contradictory at first glance. Because chemotherapy-induced bone marrow suppression reduces ANC to a critical level, the NLR and SII calculations, which include neutrophil count in the numerator, are mathematically suppressed. Therefore, low NLR, which indicates increased risk in the general sepsis population, is a direct reflection of bone marrow failure and profound neutropenia in the febrile neutropenic emergency population [10,11].

High collinearity among the parameters included in our study was evaluated, and very strong positive correlations were detected between CRP and CAR and between NLR and SII. To prevent the disruptive effect of multicollinearity on regression models, the clinically more comprehensive CAR and NLR parameters were included in the model, and the redundant parameters that would reduce the model’s success were eliminated.

Following backward stepwise multivariate logistic regression analysis and backward elimination, body temperature at admission to the ED (*p* < 0.001), serum Na level (*p* < 0.001), cellular NLR (*p* < 0.001), and clinical SI (*p* = 0.008) were identified as independent predictors of febrile neutropenia. Biomarkers may play a critical role in this clinical picture, and these parameters can be positioned as additional supporting tools to existing clinical decision-making rules or as a primary risk stratification and screening tool similar to the D-Dimer test in the algorithm for pulmonary embolism [16,17,18].

In our study, a lower NLR was observed in patients who developed febrile neutropenia. This inverse relationship should be interpreted cautiously. In the broader literature on sepsis and intensive care, a high NLR is generally considered an indicator of adverse clinical outcomes, reflecting hyperinflammation and systemic stress [19,20]. However, in chemotherapy-induced myelosuppression, the primary driver of a low NLR is the significant decline in absolute neutrophil count (ANC)—the parameter that defines the primary endpoint threshold (ANC < 1000/µL). Consequently, in this clinical scenario, the NLR partially incorporates the definition of the endpoint itself—a phenomenon referred to in the literature as “mathematical coupling” or “incorporation bias” [21]. Rather than representing a novel immunological mechanism, a low NLR primarily reflects the severity of bone marrow suppression. Future prospective studies evaluating inflammatory markers in neutropenic patients should account for this structural constraint—for instance, by focusing on baseline values prior to chemotherapy or by examining immune indicators independent of neutrophils.

A key methodological consideration in our study is the potential for optimism bias inherent in variable selection from a single dataset. To address this, we performed bootstrap resampling (1000 iterations), which yielded robust optimism-corrected discrimination and calibration metrics. Nevertheless, direct prospective head-to-head benchmarking against established clinical decision tools, such as the MASCC risk index and CISNE score, was constrained by the retrospective nature of certain qualitative ED clinical documentation. Future prospective ED studies should evaluate whether incorporating [our proposed index/markers] alongside MASCC or CISNE improves net clinical benefit and risk stratification accuracy.

The high 24-fold OR score (aOR: 24.010) of SI in the multivariate model clearly reveals the critical role of subclinical hemodynamic instability and latent tissue perfusion impairment in febrile neutropenia. The development of hyponatremia, on the other hand, can be explained by the increased secretion of antidiuretic hormone (ADH) and inflammatory cytokine load in sepsis and stands out as a strong independent risk factor.

In the ROC analysis of this multivariate integrated regression model, the AUC value was determined as 0.906 (95% CI: 0.874–0.937). At the optimal cut-off value of 0.702 determined for the model, the sensitivity and specificity of the system in distinguishing febrile neutropenia were found to be 77.4 and 87.0%, respectively; the overall diagnostic accuracy was also determined to be 80.9%. Therefore, the strongest clinical outcome of our study is that in oncology patients receiving chemotherapy and presenting to the ED, an increase in body temperature at admission, hyponatremia, paradoxically low cellular NLR levels, and a high baseline SI are strong and independent predictors of febrile neutropenia and associated adverse clinical outcomes, such as development of septic shock and need for ICU admission. This high diagnostic power suggests that instead of relying on a single laboratory value in the ED, the combination of hemodynamic (SI), vital (fever), electrolyte (Na), and cellular immune response (NLR) parameters can be a useful complementary guide in clinical decision-making processes [4,5].

The strong prognostic performance of SI and simple, calculable biomarkers included in our study can be easily evaluated by ED physicians. Such a practical approach will help avoid aggressive overloads during triage and risk stratification steps, as well as during antibiotic administration and fluid resuscitation, and ensure patients receive the right intervention at the right time by shedding light on early ICU admissions.

In our cohort, 57.7% of patients with febrile neutropenia received intravenous antibiotics during their emergency department (ED) stay, and about one-third were treated within the recommended first hour. This finding highlights a significant clinical quality gap relative to international guidelines (e.g., ASCO/IDSA), which recommend initiating broad-spectrum antibiotic therapy within 60 min of presentation [9]. Several factors may account for this observation. First, some patients may have already been receiving prophylactic or empirical oral antimicrobial therapy as outpatients before arriving in the ED. Second, operational delays during ED triage—specifically, waiting for confirmation of neutropenia via an automated complete blood count (CBC) before initiating intravenous regimens—delayed administration. Finally, limited retrospective documentation may have led to underreporting of medications administered immediately upon arrival in the department. These findings underscore an urgent need for protocolized ED triage pathways, such as rapid point-of-care CBC testing or nurse-led empirical fever protocols for oncology patients to optimize “door-to-antibiotic time.”

Although the shock index emerged as a predictor in our multivariable analysis with a notable odds ratio, we must interpret this finding with caution. The exceptionally wide confidence interval (95% CI: 1.25 (1.09–1.43)) indicates substantial statistical imprecision, likely secondary to sample size constraints and variance within the subset. Rather than establishing a definitive causal link or a standalone diagnostic trigger, the shock index in this context should be viewed as an exploratory indicator that warrants further evaluation in larger, prospective cohorts.

Among the limitations of our study are the single-center and retrospective design and the dependence of several clinical variables on data in HIMS. Furthermore, because the study period coincided with the global COVID-19 pandemic, although we aimed to exclude viral neutropenia, we could not completely rule out subclinical viral infections. Finally, since no formal internal or external validation analysis was performed on this mathematical model, this tool should be considered a potential “pre-screening support” for optimizing empirical processes while awaiting CBC results in ED triage, rather than a definitive diagnostic tool.

## 5. Conclusions

In oncology patients with prior chemotherapy exposure presenting to the emergency department, elevated admission body temperature, hyponatremia, paradoxically lower cellular neutrophil-to-lymphocyte ratios, and elevated baseline shock index serve as strong independent predictors of febrile neutropenia and associated adverse clinical outcomes, including septic shock and intensive care unit admission; accordingly, the multivariable regression model integrating these four readily accessible parameters provides a low-cost, rapidly deployable, and highly accurate decision-support framework to streamline emergency department triage and minimize time-to-treatment delays for empirical protocols. Nevertheless, given the single-center, retrospective design and preliminary nature of these findings, these results should not be interpreted as establishing direct causal relationships or immediate clinical practice guidelines but rather as an exploratory risk stratification model that warrants rigorous internal calibration and multicenter external validation prior to safe implementation in routine emergency care.

## Figures and Tables

**Table 1:** Demographic and clinical characteristics of the study population.

Variable	Febrile Neutropenia Group (*n* = 213)	Control Group (*n* = 125)	*p*-Value
Age (years)	68.00 (56.00–74.00)	68.00 (58.00–74.00)	0.711
Males, *n* (%)	101 (47.4)	57 (45.6)	0.833
Antibiotic therapy initiated, *n* (%)	123 (57.7)	26 (20.8)	<0.001
Hospital admission, *n* (%)	139 (65.3)	66 (52.8)	0.032
ICU admission, *n* (%)	48 (22.5)	13 (10.4)	0.008
Septic shock, *n* (%)	45 (21.1)	11 (8.8)	0.005

ICU: intensive care unit.

**Table 2:** Comparison of vital signs between the febrile neutropenia and control groups.

Variable	Febrile Neutropenia Group (*n* = 213)	Control Group (*n* = 125)	*p*-Value
Body temperature (°C)	37.10 (36.50–38.10)	36.70 (36.30–37.10)	<0.001
SBP (mmHg)	122.59 ± 20.74	129.60 ± 20.01	0.002
LAC (mmol/L)	1.70 (1.30–2.42)	1.50 (1.00–2.20)	0.014
Blood pH	7.39 (7.35–7.45)	7.41 (7.37–7.45)	0.049

LAC: lactate, pH: power of hydrogen, SBP: systolic blood pressure.

**Table 3:** Comparison of laboratory findings between the febrile neutropenia and control groups.

Variable	Febrile Neutropenia Group (*n* = 213)	Control Group (*n* = 125)	*p*-Value
Na (mmol/L)	134.00 (131.00–138.00)	136.00 (134.00–138.00)	0.005
CRP (mg/L)	83.50 (25.60–154.45)	41.50 (13.25–100.50)	<0.001
RBC (×10^6^/µL)	3.40 (2.84–4.02)	3.80 (3.40–4.24)	<0.001
WBC (×10^3^/µL)	1.37 (0.74–2.28)	6.26 (4.53–10.40)	<0.001
PLT (×10^3^/µL)	92.00 (43.00–155.00)	163.00 (91.00–266.00)	<0.001
NEU (×10^3^/µL)	0.44 (0.14–0.78)	4.80 (3.03–7.59)	<0.001
LYMPH (×10^3^/µL)	0.59 (0.31–1.05)	0.94 (0.54–1.44)	<0.001
MONO (×10^3^/µL)	0.17 (0.05–0.37)	0.50 (0.31–0.73)	<0.001
PCT (µg/L)	1.18 (0.30–10.98)	0.44 (0.15–3.40)	0.008

CRP: C-reactive protein, LYMPH: lymphocyte, MONO: monocyte, Na: sodium, NEU: neutrophil, PCT: procalcitonin, PLT: platelet, RBC: erythrocyte, WBC: leukocyte.

**Table 4:** Comparison of inflammatory indices between the febrile neutropenia and control groups.

Variable	Febrile Neutropenia Group (*n* = 213)	Control Group (*n* = 125)	*p*-Value
NLR	0.64 (0.22–1.33)	5.28 (2.53–11.23)	<0.001
PLR	141.43 (74.03–300.93)	176.41 (99.73–279.65)	0.220
LMR	3.76 (1.76–8.00)	1.96 (1.22–2.74)	<0.001
SII	48.13 (18.03–130.23)	897.18 (336.23–1988.69)	<0.001
CAR	27.78 (8.67–62.75)	14.25 (4.21–38.77)	0.002
FAR	116.57 (83.85–168.64)	120.91 (63.73–139.16)	0.138
LAR	0.58 (0.38–0.83)	0.50 (0.34–0.67)	0.015
SI	0.77 (0.63–0.93)	0.71 (0.59–0.85)	0.010

CAR: C-reactive protein-to-albumin ratio, FAR: fibrinogen-to-albumin ratio, LAR: lactate-to-albumin ratio, LMR: lymphocyte-to-monocyte ratio, NLR: neutrophil-to-lymphocyte ratio, PLR: platelet-to-lymphocyte ratio, SI: shock index, SII: systemic immune–inflammation index.

**Table 5:** Multivariable logistic regression analysis of independent predictors of febrile neutropenia.

Variable	B	SE	aOR (95% Cl)	*p*
Body temperature (°C)	0.811	0.206	2.25 (1.50–3.37)	<0.001
Na (mmol/L)	−0.141	0.029	0.87 (0.82–0.92)	<0.001
NLR	−0.468	0.098	0.63 (0.52–0.76)	<0.001
SI	0.224	0.069	1.25 (1.09–1.43)	0.001

CI: confidence interval, Na: sodium, NLR: neutrophil-to-lymphocyte ratio, OR: odds ratio, SI: shock index.

**Table 6:** Diagnostic performance of the final multivariable logistic regression model.

Statistics	Value
Apparent AUC (95% CI)	0.903 (0.871–0.932)
Optimism-corrected AUC	0.898
Apparent Brier score	0.122
Optimism-corrected Brier score	0.127
Mean bootstrap calibration slope	0.935
Sensitivity (threshold = 0.50)	91.55% (195/213)
Specificity (threshold = 0.50)	65.32% (81/124)
Positive predictive value (PPV)	81.93% (195/238)
Negative predictive value (NPV)	81.82% (81/99)

AUC: area under the curve; CI: confidence interval. For AUC, the 95% CI of the apparent model is presented; internal validation is based on 350 patient-level bootstrap resamplings. The performance threshold value is 0.50.

**Table 7:** Variables associated with clinical outcomes in the FN group.

Outcome/Variable	aOR (95% CI)	*p*
**Intensive care unit admission (48/213)**		
Body temperature (1 °C)	1.01 (0.74–1.39)	0.929
Sodium (1 mmol/L)	0.98 (0.92–1.04)	0.446
NLR (1 unit)	1.08 (0.96–1.23)	0.205
Shock index (0.10 units)	1.16 (1.03–1.31)	0.017
**Septic shock (45/213)**		
Body temperature (1 °C)	1.00 (0.72–1.40)	0.991
Sodium (1 mmol/L)	0.96 (0.90–1.02)	0.184
NLR (1 unit)	1.08 (0.95–1.22)	0.260
Shock index (0.10 units)	1.20 (1.05–1.36)	0.005
**30-day mortality (42/213)**		
Body temperature (1 °C)	1.09 (0.79–1.50)	0.589
Sodium (1 mmol/L)	0.97 (0.90–1.03)	0.313
NLR (1 unit)	1.02 (0.90–1.15)	0.766
Shock index (0.10 units)	1.23 (1.08–1.41)	0.002

**Table 8:** Distributions of risk scores.

Score	*n*	Mean ± SD/*n* (%)	Median (Q1–Q3)	Min–Max
MASCC total score	157	18.52 ± 3.98	19.00 (16.00–21.00)	7.00–26.00
MASCC low risk (≥21 points)	157	56 (35.67)	—	—
MASCC high risk (<21 points)	157	101 (64.33)	—	—
CISNE—assuming no mucositis ^1^	100	3.15 ± 1.83	3.00 (1.00–4.00)	0.00–6.00
CISNE—assuming presence of mucositis ^1^	100	4.15 ± 1.83	4.00 (2.00–5.00)	1.00–7.00

MASCC: 157 febrile neutropenia (FN) presentations involved malignancy, while the other 56 FN presentations had no recorded malignancy. For the CISNE model, 100 FN presentations—coded exclusively as solid tumors and involving chemotherapy—were selected as candidates. ^1^ Because there is no record of mucositis, the actual score for the two scenarios remains unknown; furthermore, clinical validation using ECOG performance status and an assessment of stability at initial presentation are also missing. Consequently, the CISNE criteria should not be considered a valid prognostic risk classification.

**Table 9:** MASCC risk classes and clinical outcomes.

Outcome	MASCC < 21 (*n* = 101)	MASCC ≥ 21 (*n* = 56)	AUC
Intensive care unit admission	40/101 (39.60%)	0/56 (0.00%)	0.883
Septic shock	39/101 (38.61%)	0/56 (0.00%)	0.850
30-day mortality	35/101 (34.65%)	2/56 (3.57%)	0.805

**Note.** Analysis includes 157 FN presentations with underlying malignancy.

## Data Availability

The data supporting the findings of this study are available within the article.

## References

[1] Meidani M., Rostami M., Moulaee S. (2012). Blood culture in neutropenic patients with fever. Int. J. Prev. Med..

[2] Rothberg B.E.G., Quest T.E., Yeung S.J., Pelosof L.C., Gerber D.E., Seltzer J.A., Bischof J.J., Thomas C.R., Akhter N., Mamtani M. (2022). Oncologic emergencies and urgencies: A comprehensive review. CA Cancer J. Clin..

[3] Kumar A., Roberts D., Wood K.E., Light B., Parrillo J.E., Sharma S., Suppes R., Feinstein D., Zanotti S., Taiberg L. (2006). Duration of hypotension before initiation of effective antimicrobial therapy is the critical determinant of survival in human septic shock. Crit. Care Med..

[4] Klastersky J., Paesmans M., Rubenstein E.B., Boyer M., Elting L., Feld R., Gallagher J., Herrstedt J., Marty M., Talcott J. (2000). The Multinational Association for Supportive Care in Cancer risk index: A multinational scoring system for identifying low-risk febrile neutropenic cancer patients. J. Clin. Oncol..

[5] Coyne C.J., Le V., Brennan J.J., Castillo E.M., Shatsky R.A., Vilke G.M. (2017). Application of the MASCC and CISNE risk-stratification scores to identify low-risk febrile neutropenic patients in the emergency department. Ann. Emerg. Med..

[6] Hu B., Yang X.R., Xu Y., Sun Y.F., Sun C., Guo W., Zhang X., Wang Z., Huang X.W., Zhou J. (2021). Systemic immune-inflammation index predicts prognosis of patients with sepsis. Front. Immunol..

[7] Ranzani O.T., Zampieri F.G., Forte D.N., Azevedo L.C.P., Park M. (2013). C-reactive protein/albumin ratio predicts 90-day mortality of septic patients. PLoS ONE.

[8] Freifeld A.G., Bow E.J., Sepkowitz K.A., Boeckh M.J., Ito J.I., Mullen C.A., Raad I.I., Rolston K.V., Young J.-A.H., Wingard J.R. (2011). Clinical practice guideline for the use of antimicrobial agents in neutropenic patients with cancer: 2010 update by the Infectious Diseases Society of America. Clin. Infect. Dis..

[9] Taplitz R.A., Kennedy E.B., Bow E.J., Crews J., Gleason C., Hawley D.K., Langston A.A., Nastoupil L.J., Rajotte M., Rolston K. (2018). Outpatient management of fever and neutropenia in adults with cancer: ASCO plus IDSA guideline update. J. Clin. Oncol..

[10] Herbst C., Naumann F., Kruse E.-B., Monsef I., Bohlius J., Schulz H., Engert A. (2009). Prophylactic antibiotics or G-CSF for the prevention of infections and improvement of survival in cancer patients undergoing chemotherapy. Cochrane Database Syst. Rev..

[11] Smith T.J., Khatcheressian J., Lyman G.H., Ozer H., Armitage J.O., Balducci L., Bennett C.L., Cantor S.B., Crawford J., Cross S.J. (2006). 2006 Update of recommendations for the use of white blood cell growth factors: An evidence-based clinical practice guideline. J. Clin. Oncol..

[12] Lilienfeld-Toal M., Dietrich M.P., Glasmacher A., Lehmann L., Breig P., Hahn C., Schmidt-Wolf I.G.H., Marklein G., Schroeder S., Stuber F. (2004). Markers of bacteremia in febrile neutropenic patients with hematological malignancies: Procalcitonin and IL-6 are more reliable than C-reactive protein. Eur. J. Clin. Microbiol. Infect. Dis..

[13] Lyman G.H., Michels S.L., Reynolds M.W., Barron R., Tomic K.S., Yu J. (2010). Risk of mortality in patients with cancer who experience febrile neutropenia. Cancer.

[14] Coyne C.J., Castillo E.M., Shatsky R.A., Chan T.C. (2022). Procalcitonin as a Predictive Tool for Death and ICU Admission among Febrile Neutropenic Patients Visiting the Emergency Department. Medicina.

[15] Gharipour A., Razavi R., Gharipour M., Mukasa D. (2020). Lactate/albumin ratio as a predictor of mortality in septic patients: A systematic review and meta-analysis. Am. J. Emerg. Med..

[16] Linkins L.-A., Lapner S.T. (2017). Review of D-dimer testing: Good, Bad, and Ugly. Int. J. Lab. Hematol..

[17] Righini M., Robert-Ebadi H., Le Gal G. (2017). Diagnosis of acute pulmonary embolism. J. Thromb. Haemost..

[18] Crawford F., Andras A., Welch K., Sheares K., Keeling D., Chappell F.M. (2016). D-dimer test for excluding the diagnosis of pulmonary embolism. Cochrane Database Syst. Rev..

[19] Ljungström L., Brinkhäll G., Eriksson B.-M., Blomqvist C., Carlsson P.-O., Hagel S., Holmqvist B., Lindquist C.-M., Nellgård B., Unge C. (2017). Diagnostic accuracy of biomarkers in identifying sepsis in the emergency department. BMC Infect. Dis..

[20] Zahorec R. (2021). Neutrophil-to-lymphocyte ratio, severe sepsis and ethical considerations. Emerg. Med. J..

[21] Forget P., Khalifa C., Defour J.-P., Latinne D., Van Pel M.-C., De Kock M. (2017). What is the normal value of the neutrophil-to-lymphocyte ratio?. BMC Res. Notes.

